# Факторы риска развития метастатической феохромоцитомы/параганглиомы

**DOI:** 10.14341/probl13331

**Published:** 2023-10-04

**Authors:** Д. В. Реброва, О. И. Логинова, С. Л. Воробьев, Н. В. Ворохобина, Е. С. Козорезова, Ф. А. Индейкин, Т. В. Савельева, И. В. Слепцов, Р. А. Черников, Е. А. Федоров, А. А. Семенов, И. К. Чинчук, Ш. Ш. Шихмагомедов, М. А. Алексеев, Л. М. Краснов, В. Ф. Русаков

**Affiliations:** Санкт-Петербургский государственный университет, Клиника высоких медицинских технологий им. Н.И. Пирогова; Санкт-Петербургский государственный университет, Клиника высоких медицинских технологий им. Н.И. Пирогова; Национальный клинический центр морфологической диагностики; Северо-Западный государственный медицинский университет им. И.И. Мечникова; Национальный клинический центр морфологической диагностики; Национальный клинический центр морфологической диагностики; Санкт-Петербургский государственный университет, Клиника высоких медицинских технологий им. Н.И. Пирогова; Санкт-Петербургский государственный университет, Клиника высоких медицинских технологий им. Н.И. Пирогова; Санкт-Петербургский государственный университет, Клиника высоких медицинских технологий им. Н.И. Пирогова; Санкт-Петербургский государственный университет, Клиника высоких медицинских технологий им. Н.И. Пирогова; Санкт-Петербургский государственный университет, Клиника высоких медицинских технологий им. Н.И. Пирогова; Санкт-Петербургский государственный университет, Клиника высоких медицинских технологий им. Н.И. Пирогова; Санкт-Петербургский государственный университет, Клиника высоких медицинских технологий им. Н.И. Пирогова; Санкт-Петербургский государственный университет, Клиника высоких медицинских технологий им. Н.И. Пирогова; Санкт-Петербургский государственный университет, Клиника высоких медицинских технологий им. Н.И. Пирогова; Санкт-Петербургский государственный университет, Клиника высоких медицинских технологий им. Н.И. Пирогова

**Keywords:** феохромоцитома, параганглиома, метастатическая феохромоцитома/параганглиома, злокачественная феохромоцитома/параганглиома, шкала PASS, шкала GAPP

## Abstract

В настоящее время все феохромоцитомы/параганглиомы (ФЕО/ПГ) считаются злокачественными, поскольку обладают метастатическим потенциалом. ФЕО/ПГ разделяют на «метастатические» и «неметастатические». Метастатические ФЕО/ПГ могут быть с синхронным метастазированием (метастазы появляются одновременно с выявленной первичной опухолью) или метасинхронным (метастазы развиваются после удаления первичной опухоли). Термин метастатическая ФЕО/ПГ не применяется при наличии инвазии опухоли в окружающие органы и ткани, без наличия отдаленных метастазов лимфогенного или гематогенного происхождения.

Принято считать, что около 10% ФЕО и около 40% симпатических ПГ обладают метастатическим потенциалом. В среднем распространенность ФЕО/ПГ с наличием метастазов составляет 15-20%.

В литературе широко обсуждаются факторы риска метастатической ФЕО/ПГ, наиболее значимыми из которых считаются группы клинических, морфологических и генетических признаков. В обзоре представлено обсуждение таких факторов риска метастатической ФЕО/ПГ как возраст, локализация и тип гормональной секреции опухоли, размер и характер роста новообразования, наличие некроза и инвазии в сосуды, капсулу опухоли, окружающую жировую клетчатку, высокая клеточность и митотическая активность, индекс Ki-67, экспрессия хромогранина В и белка S100, наличие генетических мутаций из трех основных кластеров (псевдогипоксии, киназного сигналинга и сигналинга Wnt).

На протяжении последних двух десятилетий рядом авторов предложены различные предикторные факторы и шкалы для оценки вероятности развития метастатической формы ФЕО/ПГ. В обзоре подробно представлено описание и сравнение чувствительности и специфичности предикторных шкал PASS, GAPP, M-GAPP, ASES и COPPS.

## Abstract (en)

Currently, all pheochromocytoma/paraganglioma (PPGLs) are considered malignant due to metastatic potential. Consequently, PPGLs are divided into «metastatic» and «non-metastatic». Metastatic PPGLs can be with synchronous metastasis (metastases appear simultaneously with the identified primary tumor) or metachronous (metastases develop after removal of the primary tumor). The term metastatic PPGLs is not used in the presence of tumor invasion into surrounding organs and tissues, without the presence of distant metastases of lymphogenic or hematogenic origin. It is generally believed that about 10% of pheochromocytomas and about 40% of sympathetic paragangliomas have metastatic potential. On average, the prevalence of PPGLs with the presence of metastases is 15–20%. Risk factors for metastatic PPGLs are widely discussed in the literature, the most significant of which are groups of clinical, morphological and genetic characteristics. The review presents a discussion of such risk factors for metastatic PPGLs as age, localization and type of hormonal secretion of the tumor, the size and growth pattern of the adrenal lesion, the presence of necrosis and invasion into the vessels, the tumor capsule surrounding adipose tissue, high cellular and mitotic activity, Ki-67 index, expression of chromogranin B and S100 protein, the presence of genetic mutations of three main clusters (pseudohypoxia, kinase signaling and Wnt signaling).

Over the past two decades, a number of authors have proposed various predictor factors and scales for assessing a probability of metastatic PPGLs. The review contains detailed description and comparison of sensitivity and specificity of such predictor scales as PASS, GAPP, M-GAPP, ASES and COPPS.

Феохромоцитома (ФЕО) — опухоль, развивающаяся из хромаффинной ткани мозгового слоя надпочечников, параганглиома (ПГ) — опухоль из симпатических или парасимпатических ганглиев [1]. Клинические проявления и морфологическая структура опухолей идентичны. Наиболее частым симптомом ФЕО/ПГ является повышение уровня артериального давления. Возможно как пароксизмальное течение артериальной гипертензии с развитием симпатоадреналовых кризов, так и вариант с постоянной артериальной гипертензией, устойчивой к антигипертензивной терапии. Около 10% ФЕО/ПГ протекают бессимптомно [2]. Подобные опухоли чаще всего являются случайными находками при лучевых исследованиях и требуют более углубленного обследования пациентов для определения характера инциденталомы с целью оценки необходимости проведения операции и предоперационной подготовки [3].

Деление ФЕО на доброкачественную и злокачественную, сохранявшееся до 4 пересмотра классификации ВОЗ (2017 г.), стало неактуальным. В настоящее время все ФЕО/ПГ считаются по определению злокачественными, поскольку обладают метастатическим потенциалом. В связи с этим ФЕО/ПГ разделяют на «метастатические» и «неметастатические» [4]. Метастатические ФЕО/ПГ могут быть с синхронным метастазированием (метастазы, или вторичные депозиты, появляются одновременно с выявленной первичной опухолью) или с метахронным (метастазы развиваются после удаления первичной опухоли) [5]. Термин «метастатическая ФЕО/ПГ» не применяется при наличии инвазии опухоли в окружающие органы и ткани, без наличия отдаленных метастазов лимфогенного или гематогенного происхождения.

Принято считать, что около 10% ФЕО и около 40% симпатических ПГ обладают метастатическим потенциалом [6]. Парасимпатические ПГ головы и шеи метастазируют редко [7]. В среднем распространенность ФЕО/ПГ с наличием метастазов составляет 15–20% [6][8].

В большинстве случаев спорадической ФЕО/ПГ хирургическое удаление первичной опухоли приводит к полному излечению пациентов [9]. Наиболее распространенным в литературе является мнение, что в случае развития метастатического поражения 5-летняя выживаемость больных не превышает 50% [10][11]. Более оптимистичные данные получены в исследовании Hamidi O. и соавт. (2017 г.), в котором на крупной выборке пациентов показано, что 5-летняя выживаемость общая и отдельно в группе метастатической ФЕО/ПГ намного выше, чем предполагалось ранее, и составляет 85,4 и 88,2% соответственно, а 10-летняя — 72,5 и 77,9% соответственно, что демонстрирует в целом относительно благоприятный прогноз даже при распространенной форме заболевания [12]. Однако до настоящего времени нередки случаи обнаружения данной патологии лишь при аутопсии [13–16].

По данным литературы, синхронные метастазы ФЕО/ПГ выявляются в 35–50% случаев [17]. По данным оценки базы пациентов с новообразованиями надпочечников и забрюшинного пространства, прооперированных в Клинике высоких медицинских технологий им. Н.И. Пирогова СПбГУ с 2010 по 2022 гг., метастазы были выявлены лишь у 6 из 285 пациентов (2,1%) с ФЕО и абдоминальными ПГ. Метахронные вторичные депозиты могут выявляться спустя несколько лет после удаления первичной опухоли [12][17], в связи с чем Европейское общество эндокринологов рекомендует наблюдать всех больных с ФЕО/ПГ в течение 10 лет [18]. Кроме того, существует рекомендация о пожизненном наблюдении пациентов с высоким риском развития метастатической ФЕО/ПГ [18], однако до настоящего времени отсутствуют надежные способы оценки метастатического потенциала данных опухолей.

На протяжении последних двух десятилетий рядом авторов предложены различные предикторные факторы и шкалы для оценки вероятности развития метастатической формы ФЕО/ПГ. Факторы риска можно разделить на клинические, морфологические и генетические.

## КЛИНИЧЕСКИЕ ФАКТОРЫ

Данные о влиянии различных клинических факторов на агрессивность течения и появление метастазов ФЕО/ПГ разрозненны и различны. По результатам ретроспективного исследования Hamidi О. и соавт. (2017 г.), одним из значимых факторов быстрого прогрессирования заболевания с развитием летального исхода в течение 5 лет является более пожилой возраст [12]. Напротив, в работе Cho Y.Y. и соавт. (2018 г.) более высоким риском для метастазирования считался возраст пациентов до 35 лет [19], в работе Zelinka T. и соавт. (2011 г.) — до 40 лет [20].

Некоторые авторы рассматривают в качестве предиктора метастазирования характер секреции опухоли. Так, в работах Ayla-Ramirez M. и соавт. (2013 г.) и Szalat A. и соавт. (2010 г.) установлено, что метастатические ФЕО/ПГ редко секретируют адреналин, тогда как Eisenhofer G. и соавт. (2012 г.) сообщали о более характерной для данного вида опухолей выработке дофамина, а Cho Y.Y. и соавт. (2018 г.) и Zelinka T. и соавт. (2011 г.) в качестве риска метастазирования рассматривали норадреналиновый тип секреции [6][19][20][21][22].

В исследовании Hamidi O. и соавт. (2017 г.) у больных с метастатической формой ФЕО/ПГ функциональная активность была выявлена в 197 из 248 случаев. Из доступных анализу данных установлена повышенная адренергическая продукция у 61 из 177 (34,5%) пациентов, норадренергическая — у 113 из 177 (63,8%), дофаминергическая — у 71 из 177 (42%). У 51 пациента из 248 обследованных (21%) гормональная активность отсутствовала [12].

В исследовании Stenman A. и соавт. (2019 г.) получены данные об ассоциации повышенного уровня хромогранина В в плазме крови и повышения его экспрессии в опухолях с высокой оценкой по шкале PASS, что позволило предположить возможность использования данного показателя для предоперационной оценки риска метастазирования ФЕО/ПГ [23].

Многими авторами показано, что ПГ вненадпочечниковой локализации обладают более высоким метастатическим потенциалом по сравнению с ФЕО. В исследовании Ayla-Ramirez М. и соавт. (2011 г.) на крупной выборке из 371 пациента (267 ФЕО и 104 ПГ) частота вторичного поражения составила 25% при ФЕО по сравнению с 65–70% при ПГ [6]. Из 272 больных с метастатическими ФЕО/ПГ, обследованных с 1960 по 2016 гг. в клинике Мейо, 36% составили ФЕО, 58% — ПГ, 6% — одновременное наличие обоих типов опухолей [12].

## МОРФОЛОГИЧЕСКИЕ ФАКТОРЫ

Большинство авторов согласны тем, что к морфологическим признакам высокой вероятности развития метастатической ФЕО/ПГ относятся наличие некроза опухоли, диффузный характер роста новообразования, высокая клеточность, инвазия в сосуды и капсулу опухоли, а также в окружающую жировую клетчатку, высокая митотическая активность (>3 митозов на 10 полей зрения при увеличении х400).

August C. и соавт. (2004 г.) наиболее информативным фактором прогнозирования наличия метастатической опухоли считали вес ФЕО/ПГ: опухоли массой 100 г и более статистически значимо чаще оказывались метастатическими [24]. Схожие данные получены в исследовании Wailly P. и соавт. (2012 г.). Авторы получили прямую корреляцию размеров и массы опухоли с выявлением метастазов [25]. Тем не менее August C. и соавт. (2004 г.) отмечали, что у 25% пациентов со вторичным поражением выявлялись ФЕО/ПГ менее 100 г с минимальным весом в 22 г, тогда как у 80-летнего больного с образованием массой 280 г в процессе дальнейшего 8-летнего наблюдения метастазов выявлено не было [24]. В исследовании Agarwal A. и соавт. (2010 г.) было выдвинуто предположение, что размер опухоли более 6 см является прогностически неблагоприятным фактором в отношении метастатической ФЕО, хотя у 2 пациентов из исследованных 6 опухоли были размером 4 см [26]. В работе Ayla-Ramirez М. и соавт. (2011 г.) получены данные о снижении выживаемости у пациентов с размером опухоли более 5 см, однако 16% больных с метастатическими формами (3 ФЕО и 11 ПГ) имели новообразования менее 5 см. Кроме того, на примере нескольких пациентов была продемонстрирована возможность распространения заболевания даже при небольших образованиях размером 2 см с симультанными метастазами в лимфатических узлах и опухолях размером 1 см с выявленным костным метастазом через 1 год после операции [6]. В публикации Zelinka T. и соавт. (2011 г.) медиана размера ФЕО была больше в группе метастатической формы заболевания (8 см против 5,8 см), однако минимальные и максимальные размеры значимо не отличались (2,4 см против 2 см и 17 см против 16 см соответственно) [20]. В работе Hamidi O. и соавт. (2017 г.) получены данные о высокой положительной корреляционной связи большого размера опухоли с риском вторичного поражения и быстрого прогрессирования заболевания, при этом размер более 6 см рассматривался как важный независимый фактор риска. Тем не менее в данной крупной выборке описаны метастатические ФЕО размером от 3 см и ПГ — от 0,9 см [12]. Таким образом, размер и масса опухоли не могут считаться надежным прогностическим критерием метастатического распространения ФЕО/ПГ.

Рядом авторов в качестве одного из прогностических факторов метастатического распространения рассматривается наличие некроза опухоли [20][25][27].

В определении метастатического потенциала опухолей хромафинной ткани и в отношении информативности индекса пролиферативной активности мнения авторов различны. В работе Kulkarini M.M. и соавт. (2016 г.) при оценке 10 ФЕО/ПГ значение Ki-67 более 3% было установлено только у 2 из 3 метастатических ФЕО/ПГ (повышен у 2 ПГ и не повышен у 1 ФЕО), тогда как все 7 неметастатических новообразований характеризовались значением Ki-67 менее 3% [28]. В исследовании Wailly P. и соавт. (2012 г.) у всех 7 пациентов с ФЕО с установленными метастазами опухоли Ki-67 превышал 4%, однако в 1 из 46 случаев без выявленного отдаленного поражения Ki-67 составил 11% [25]. August C. и соавт. (2004 г.) проводили сравнительную геномную гибридизацию 41 ФЕО/ПГ, в результате которой не было получено достоверных данных о хромосомных аберрациях, характерных для метастатической формы заболевания. В данном исследовании установлено, что метастатические ФЕО/ПГ обладают более высокой пролиферативной активностью с индексом MIB-1 более 5%, при этом отмечена слабая мембранная экспрессия белка CD44-S [24]. Тем не менее, несмотря на то что в приведенных исследованиях показано, что описанные маркеры отражают повышенную пролиферативную активность и нарушение способности к дифференцировке клеток метастатических ФЕО/ПГ, некоторые методологические ограничения не позволяют внедрять эти показатели как убедительные в практические рекомендации.

Противоречивы данные о роли снижения количества сустентоцитов и, соответственно, экспрессии белка S100 как предиктора метастатического распространения ФЕО/ПГ. В исследовании Kulkarini M.M. и соавт. (2016 г.) из 3 случаев с отдаленным распространением опухоли у 2 пациентов иммунореактивность S100 была слабой, тогда как у 1 пациента — умеренной. В 7 неметастатических случаях экспрессия S100 была умеренной или сильной [28]. В работах Unger P. и соавт. (1991 г.) и, позднее, Wailly P. и соавт. (2012 г.) отсутствие экспрессии S100 расценивалось как один из надежных гистопатологических признаков высокого риска вторичного поражения [26][29]. Однако Bialas M. и соавт. (2013 г.) показали высокую вариабельность подсчета S100-позитивных клеток в зависимости от выбранного для участка опухолевого материала, что не позволяет отнести данный параметр к надежному стандартизированному признаку риска метастазирования ФЕО/ПГ [30]. Таким образом, применение экспрессии белка S100 как фактора риска ограничено противоречивостью результатов исследований, в том числе, вероятно, обусловленных малыми размерами представленных выборок.

## ГЕНЕТИЧЕСКИЕ ФАКТОРЫ

По современным представлениям, около 40–50% ФЕО/ПГ ассоциированы с наличием генетических мутаций, даже в отсутствие отягощенного семейного анамнеза [31]. Наследственные синдромы и известные в настоящее время генетические мутации, ассоциированные с развитием ФЕО/ПГ, могут быть разделены на 3 основные группы: приводящие к клеточной псевдогипоксии, ассоциированные с нарушениями киназного сигналинга и сигналинга Wnt [32] (табл. 1).

**Table:** Таблица 1. Группы генетических мутаций, ассоциированных с развитием феохромоцитомы/параганглиомы Table 1. Groups of genetic mutations associated with the development of pheochromocytoma/paraganglioma

Группы генетических мутаций	Гены
Приводящие к клеточной псевдогипоксии	SDHA, SDHB, SDHC, SDHD, SDHAF2, FH, VHL, EPAS1
Ассоциированные с нарушениями киназного сигналинга	RET, NF1, TMEM127, HRAS, MAX
Ассоциированные с нарушениями сигналинга Wnt	CSDE1, MAML3

Патогенез заболевания при мутациях в генах группы псевдогипоксии (SDHA, SDHB, SDHC, SDHD, SDHAF2, FH, VHL, EPAS1) связан с избыточной активацией факторов, индуцируемых гипоксией (hypoxia inducible factors, HIFs), в отсутствие гипоксии. Для новообразований данной группы характерно более агрессивное течение [32]. По данным ряда исследований установлено, что из них наиболее высоким потенциалом метастазирования (до 40–50%) обладают опухоли у пациентов с патогенным вариантом гена SDHB [6][12][33–35]. Количество сукцината в опухоли, измеряемого высокоэффективной жидкостной хроматографией — масс-спектрометрией (ВЭЖХ-МС), выше у метастатических ФЕО/ПГ по сравнению с неметастатическими, что обусловлено наличием мутаций гена SDHB [36]. При этом частота вторичного поражения при дефектах генов SDHA, SDHC и VHL относительно невысока [32]. В исследовании Ayla-Ramirez М. и соавт. (2011 г.) около половины метастатических случаев ФЕО/ПГ были ассоциированы с положительной мутацией SDHB, у большинства из них выявлялась ПГ. При этом только у одного больного из выборки 89 распространенных форм была выявлена редкая мутация SDHC [6]. Схожие данные получены в исследовании Hamidi О. и соавт. (2017 г.) по данным обследования 272 пациентов с метастатической ФЕО/ПГ, из которых у 81 (30%) были выявлены генетические синдромы, в том числе в 42 случаях (15,4% от общего числа и 52% от всех генетически детерминированных), обусловленные мутацией SDHB. В то же время мутация SDHC была выявлена лишь у 2 из 272 больных (0,7%) [12]. Для мутации генов SDHD и SDHAF2 более характерно развитие ПГ головы и шеи с низким риском метастазирования [7]. ФЕО/ПГ в рамках синдрома наследственного лейомиоматоза и почечно-клеточного рака, обусловленного инактивирующими мутациями гена фумаратгидратазы (FH), встречается менее чем в 1% случаев, однако из них более 50% протекают агрессивно с метастазированием [32].

Мутации киназного сигналинга (RET, NF1, TMEM127, HRAS, MAX) приводят к изменению активности таких киназных сигнальных путей, как RAS/RAFG/MAPK и PI3K/AKT/mTOR, что сопровождается возникновением и прогрессией опухоли [32]. Для этого типа неоплазии характерны мультифокальность поражения и высокая частота рецидивирования [37]. При наследственных синдромах в данной группе описаны случаи метастатического распространения ФЕО/ПГ, однако частота их возникновения крайне редка.

Гены CSDE1 и MAML3 приводят к онкогенезу путем активации сигнальных путей Wnt и Hedgehog [32]. Исследования данных мутаций немногочисленны. Считается, что новообразования этой группы характеризуются наиболее агрессивным течением, активным метастазированием, частыми рецидивами [37]. Кроме того, имеются данные об ассоциации метастатической ФЕО/ПГ с соматическими мутациями генов SETD2 и ATRX, а также с активирующей мутацией промотера TERT [38].

В связи с установленной связью распространенности метастатических ФЕО/ПГ и наличия специфических мутаций генов многие исследователи указывают на необходимость генетического тестирования всех пациентов с впервые выявленным заболеванием [39–41]. Установлено, что более позднее выполнение генетического тестирования ассоциировано с повышенным риском рецидива и снижением выживаемости [42]. При этом нельзя не учитывать высокую финансовую нагрузку на государственную систему здравоохранения при рутинном применении генетических панелей для всех пациентов с ФЕО/ПГ [43]. Принимая во внимание данный фактор, некоторые авторы рекомендуют проводить скрининг только среди определенных категорий пациентов, например, у лиц младше 20 лет, при наличии семейного анамнеза или каких-либо клинических признаков наследственного заболевания, а также у пациентов с ПГ из симпатических ганглиев [8][44].

## ПРЕДИКТОРНЫЕ ШКАЛЫ

На основании клинических, морфологических и генетических факторов были созданы различные предикторные шкалы для прогноза рисков метастазирования ФЕО/ПГ, имеющие как определенные преимущества, так и ограничения.

## ШКАЛА PASS

Балльная шкала ФЕО надпочечников (Pheochromocytoma of the Adrenal gland Scaled Score, PASS) была предложена в 2002 г. Thompson L.D.R. и стала первой прогностической системой для оценки метастатического потенциала ФЕО [45]. В своей работе Thompson L.D.R. проводил оценку морфологической картины 100 удаленных ФЕО пациентов с 10-летней историей наблюдения после операции: 50 — с доброкачественной опухолью, 50 — со злокачественными новообразованиями, при этом в последней группе у 33 были выявлены отдаленные метастазы [45].

Шкала PASS включает 12 гистологических признаков, каждый из которых оценивается в 1 или 2 балла; максимальное количество баллов — 20. При сумме баллов 4 и более потенциал злокачественности опухоли оценивается как высокий.

Исследуемые критерии:

Несмотря на то, что система PASS разрабатывалась для оценки злокачественного потенциала ФЕО, в последующих исследованиях была показана ее эффективность и при оценке ПГ [10][46][47].

В ряде исследований проводилась оценка диагностической точности системы PASS, однако результаты их оказались противоречивыми. Стоит отметить, что до пересмотра морфологической классификации ВОЗ 2017 г. термин «злокачественная ФЕО» применялся не только для обозначения метастатических форм, в связи с чем сравнение результатов исследований разных лет представляется затруднительным. Так, как отмечалось выше, по данным динамического наблюдения Thompson L.D.R. (2002 г.) группы анализа злокачественных ФЕО, на основании которой разработана шкала PASS, метастатическая форма установлена только у 33 пациентов из 50 [45].

В работе August C. и соавт. (2004 г.) проанализированы 43 случая ФЕО/ПГ, оцененные по шкале PASS как злокачественные: 37 образований надпочечниковой и 6 вненадпочечниковой локализации. В процессе динамического наблюдения у 1 пациента был диагностирован синдром МЭН 2А, у 20 больных выявлены метастазы. Таким образом, в описываемой выборке чувствительность метода составила 100%, тогда как специфичность – 0% [24]. Напротив, хорошие результаты получены в исследовании Szalat и соавт. (2010 г.) при оценке 16 случаев метастатической ФЕО/ПГ, из которых у 8 пациентов был доступен гистологический материал для анализа: 7 ФЕО и 1 ПГ. В 7 случаях из пересмотренных опухолей по шкале PASS было получено более 4 баллов, тогда как в одном случае ФЕО — менее 4 баллов, при этом чувствительность метода составила 87,5%. При анализе 19 новообразований без выявленных метастазов в течение 5-летнего наблюдения по шкале PASS все ФЕО оказались со значениями менее 4 баллов и со специфичностью 100%. Однако авторы сообщают о возможной низкой воспроизводимости ряда морфологических параметров, таких как ядерная гиперхромия и выраженный клеточный полиморфизм, в связи с чем в этой работе пересмотр всех гистологических препаратов осуществлялся одним специалистом [21].

Более убедительными можно считать данные метаанализа крупной выборки из 809 ФЕО, в котором 102 из 105 злокачественных образований (включающих в себя в разных публикациях как ФЕО с диагностированным метастатическим распространением, так и с прорастанием в соседние органы и ткани, а также с рецидивом опухоли) оценены по шкале PASS в 4 и более баллов с чувствительностью 97%. Анализ показал довольно низкую специфичность метода в 68%, так как из 704 неметастатических ФЕО лишь в 480 случаях было установлено менее 4 баллов [46].

Kulkarini M.M. и соавт. (2016 г.) представили данные 4 случаев ПГ. У 2 пациентов были выявлены вторичные очаги с оценкой чувствительности и специфичности шкалы PASS в 100% [28]. В метаанализе Stenman A. и соавт. (2019 г.) получены менее оптимистичные результаты анализа 42 ПГ, из которых у 13 метастатических опухолей сумма баллов равнялась или превышала 4 (чувствительность 100%), однако при доброкачественном течении заболевания такой же результат был получен у 8 из 29 пациентов, а у 21 из 29 обследованных сумма баллов была менее 4 (специфичность 72%) [46]. Еще более низкие результаты получены в исследовании Agarwal A. и соавт. (2010 г.), в котором PASS >4 баллов было выявлено у 5 из 6 пациентов с метастазами ФЕО (чувствительность 83%) и у 27 из 84 неметастатических случаев (специфичность 67,9%) [26].

По мнению многих авторов, применение гистологической шкалы PASS ограничено в связи с низким уровнем воспроизводимости морфологических признаков по данным разных исследований [10][46][47].

## ШКАЛА GAPP

Система стадирования для ФЕО и ПГ (Grading System for Adrenal Pheochromocytoma and Paraganglioma, GAPP) предложена Kimura N. и соавт. в 2014 г. [48]. Модель GAPP основана на алгоритме PASS с существенными изменениями: исключение наиболее вариабельных гистологических параметров с сохранением только 4 критериев (гистологический паттерн, клеточность, наличие комедо-некрозов, инвазия в капсулу и сосуды), добавление иммуногистохимических параметров (индекс пролиферации Ki-67) и клинических характеристик (лабораторные данные — тип секреции катехоламинов). В отличие от шкалы PASS, диагностическая система GAPP разработана как для ФЕО, так и для ПГ. По предложению авторов, на основании результатов оценки характеристик все опухоли ФЕО/ПГ разделены на три группы: высокодифференцированные (0–2 балла), с умеренной дифференцировкой (3–6 баллов) и низкодифференцированные (7–10 баллов). Предполагается, что высокодифференцированные опухоли обладают меньшим метастатическим потенциалом и лучшей общей выживаемостью, однако даже в оригинальном исследовании специфичность данной градации составила 96%: у 4 из 111 пациентов были выявлены метастазы, при этом 5-летняя выживаемость составила 100%. Метастатическое распространение было выявлено у 21 из 35 больных с ФЕО/ПГ (60%) с умеренной дифференцировкой и у 15 из 17 пациентов (88%) с высокодифференцированными образованиями с 5-летней выживаемостью 67 и 22% соответственно [48].

По данным метаанализа Stenman A. и соавт. (2019 г.), 175 случаев ФЕО, из которых 4 были расценены как злокачественные (с наличием метастазов или с местным рецидивом), чувствительность применения системы GAPP составила 50%: у 2 пациентов опухоль оценена в 3 и более баллов, тогда как у 2 — менее 3 баллов. В 35 неметастатических случаях по шкале GAPP был получен результат 3 и более баллов, тогда как 136 образований получили менее 3 баллов, что определило специфичность 80%. Более высокие показатели 100% чувствительности прогностического алгоритма были получены для ПГ: все 4 включенных в анализ метастатических случая оценены в 3 и более баллов. Тем не менее специфичность шкалы GAPP для данной выборки составила 68%, так как 10 из 31 и 20 из 31 неметастатических ПГ получили ≥3 и <3 баллов соответственно [46]. В работе Бритвина Т.А. и соавт. (2021 г.) получена значимая положительная корреляция между системами PASS и GAPP, а также обеих шкал с размером ФЕО [49].

## МОДИФИЦИРОВАННАЯ СИСТЕМА M-GAPP

Модифицированная шкала для ФЕО и ПГ (M-GAPP) была предложена Koh J.M. и соавт. (2017 г.) [50]. К критериям балльной системы GAPP был добавлен такой важный прогностический фактор, как наличие в опухолевых клетках мутации гена SDHB. Однако диагностическая точность этой шкалы не стала более высокой: метастатическими опухолями оказались 12 из 34 (35,3%) с оценкой ≥4 баллов по PASS, 12 из 40 (30%) умеренно- или низкодифференцированных по GAPP и 10 из 19 (52,6%) с оценкой ≥3 баллов по M-GAPP [50]. По данным метаанализа Wang Y. и соавт. (2020 г.), чувствительность шкалы M-GAPP составила 67%, специфичность — 84%, что оказалось даже ниже аналогичной оценки по системе GAPP [10].

## ШКАЛА ASES

Cho Y.Y. и соавт. (2018 г.) на основании оценки данных 333 пациентов (305 ФЕО и 28 ПГ), у 23 из которых были выявлены метастазы (18 ФЕО и 5 ПГ), предложили клиническую прогностическую шкалу оценки метастатического потенциала ASES — сокращенное от age (возраст), size (размер), extra-adrenal (вненадпочечниковая локализация), secretory (гормональная секреция) [19]. Возраст до 35 лет включительно, размер опухоли 6 см и более, вненадпочечниковая локализация и норадреналиновый тип секреции по данной шкале оцениваются по 1 баллу. При получении результата 2 и более баллов чувствительность данной шкалы составила 61% (14/23), специфичность — 80% (248/310). 10-летняя выживаемость пациентов с оценкой ≥2 баллов составила 30%, <2 — 86% [19]. Несмотря на низкую диагностическую точность данной шкалы, возможность прогностической оценки метастатического потенциала опухоли позволяет говорить о важности учета клинических признаков, а не только гистопатологических характеристик ФЕО/ПГ [10].

## ШКАЛА COPPS

Прогностическая шкала композитной ФЕО/ПГ (Composite Pheochromocytoma/paraganglioma Prognostic Score, COPPS), предложенная в 2019 г. Pierre C. и соавт., включает три клинико-патологических характеристики (размер опухоли, наличие некроза и сосудистой инвазии) и иммуногистохимические характеристики (утрата экспрессии S100 и SDHB) [27]. В данном исследовании оценивались данные 147 пациентов (107 ФЕО и 40 ПГ), из которых у 9 были выявлены метастазы (2 ФЕО и 7 ПГ). По результатам анализа установлена статистически значимая положительная корреляция высокого риска метастатического распространения ФЕО/ПГ с такими параметрами, как вненадпочечниковая локализация опухоли, наличие мутации в гене SDHB, некроз опухоли, клеточный мономорфизм, >3 митозов в 10 полях зрения при увеличении х400, инвазии в капсулу и сосуды, утрата экспрессии генов S100 и SDHB, размер более 7 см, возраст более 40 лет, уровень экспрессии MCM6. Кроме того, совокупность большинства этих параметров коррелировали с показателями выживаемости без прогрессирования.

Из данных параметров только 5 были независимо ассоциированы с вторичным распространением ФЕО/ПГ: размер опухоли более 7 см (1 балл), сосудистая инвазия (1 балл), некрозы опухоли (5 баллов), утрата экспрессии опухолевыми клетками гена S100 (2 балла), утрата экспрессии гена SDHB (1 балл). На их основе была составлена шкала COPPS с максимальным значением в 10 баллов, по которой оценка в ≥3 баллов свидетельствует в пользу высокого метастатического потенциала опухоли. Чувствительность данной прогностической системы составила 100%, специфичность — 92,4% (табл. 2) [27].

**Table:** Таблица 2. Сравнение морфологических шкал для прогноза рисков метастазирования феохромоцитомы/параганглиомы Table 2. Comparison of morphological scales for predicting the risk of metastasis of pheochromocytoma/paraganglioma

Название шкалы	PASS	GAPP	M-GAPP	ASES	COPPS
Впервые предложена	Thompson L.D.R. (2002) [45]	Kimura N. и соавт. (2014) [48]	Koh J.M. и соавт. (2017) [50]	Cho Y.Y. и соавт. (2018) [19]	Pierre C. и соавт. (2019) [27]
Применима для феохромоцитомы	+	+	+	+	+
Применима для параганглиомы		+	+	+	+
Возраст				+	
Размер опухоли				+	+
Тип секреции катехоламинов		+	+	+	
Вненадпочечниковая локализация				+	
Морфологические критерии:					
крупные гнезда или диффузный рост клеток	+	+	+		
очаги некроза	+	+	+		+
высокая клеточность	+	+	+		
мономорфизм клеток	+				
веретенообразные клетки	+				
фигуры митоза — более 3 в 10 последовательных полях зрения	+				
атипические фигуры митоза	+				
прорастание в жировую клетчатку	+				
сосудистая инвазия	+	+	+		+
инвазия капсулы	+	+	+		
выраженный клеточный полиморфизм	+				
ядерная гиперхромия	+				
Индекс пролиферации Ki-67		+	+		
Утрата экспрессии S100					+
Мутации гена SDHB в клетках опухоли			+		+

## ЗАКЛЮЧЕНИЕ

ФЕО/ПГ признаны злокачественными опухолями, однако установление метастатического потенциала ФЕО/ПГ является сложной задачей. Предложенные разными авторами преимущественно морфологические признаки и балльные шкалы не обладают достаточно высокими чувствительностью и специфичностью для достоверного определения прогноза опухолевого заболевания. В настоящее время наиболее рациональным представляется индивидуальный подход к оценке прогноза с учетом как клинических, так и морфологических, генетических признаков. В связи с отсутствием надежных маркеров определения метастатического потенциала, согласно клиническим рекомендациям, пациенты с ФЕО/ПГ подлежат пожизненному наблюдению. Представляется, что накопление данных углубленного предоперационного обследования с целью выявления симультанных метастазов и регулярного динамического наблюдения пациентов после хирургического лечения с целью выявления отдаленных очагов вторичного распространения опухоли в сочетании с установлением наиболее специфичных морфологических признаков и генетических повреждений в будущем позволят повысить чувствительность и специфичность показателей рисков метастазирования для этой группы пациентов.

## ДОПОЛНИТЕЛЬНАЯ ИНФОРМАЦИЯ

Источники финансирования. Работа выполнена по инициативе авторов без привлечения финансирования.

Конфликт интересов. Авторы декларируют отсутствие явных и потенциальных конфликтов интересов, связанных с содержанием настоящей статьи.

Участие авторов. Все авторы внесли существенный вклад в поиск и оценку данных литературы, написание статьи или внесение в рукопись важных правок с целью повышения научной ценности статьи.

Все авторы одобрили финальную версию статьи перед публикацией, выразили согласие нести ответственность за все аспекты работы, подразумевающую надлежащее изучение и решение вопросов, связанных с точностью или добросовестностью любой части работы.
